# Stern-Judging: A Simple, Successful Norm Which Promotes Cooperation under Indirect Reciprocity

**DOI:** 10.1371/journal.pcbi.0020178

**Published:** 2006-12-29

**Authors:** Jorge M Pacheco, Francisco C Santos, Fabio A. C. C Chalub

**Affiliations:** 1 Centro de Física Teórica e Computacional and Departamento de Física da Faculdade de Ciências, Lisbon, Portugal; 2 IRIDIA, CoDE, Université Libre de Bruxelles, Brussels, Belgium; 3 Departamento de Matemática da Universidade Nova de Lisboa and Centro de Matemática e Aplicações, Caparica, Portugal; Princeton University, United States of America

## Abstract

We study the evolution of cooperation under indirect reciprocity, believed to constitute the biological basis of morality. We employ an evolutionary game theoretical model of multilevel selection, and show that natural selection and mutation lead to the emergence of a robust and simple social norm, which we call *stern-judging.* Under stern-judging, helping a good individual or refusing help to a bad individual leads to a good reputation, whereas refusing help to a good individual or helping a bad one leads to a bad reputation. Similarly for tit-for-tat and win-stay-lose-shift, the simplest ubiquitous strategies in direct reciprocity, the lack of ambiguity of stern-judging, where implacable punishment is compensated by prompt forgiving, supports the idea that simplicity is often associated with evolutionary success.

## Synopsis

Humans, unlike other animal species, form large social groups in which cooperation among non-kin is widespread. This contrasts with the general assumption that the strong and selfish individuals are the ones who benefit most from natural selection. Among the different mechanisms invoked to explain the evolution of cooperation, indirect reciprocity is associated with cooperation supported by reputation: I help you and someone else helps me. However, how did reputation evolve and which type of moral is encapsulated in those social norms that are evolutionary successful? Suggesting a simple scenario for the evolution of social norms, Pacheco, Santos, and Chalub propose a reputation-based multilevel selection model, where individual behaviour and moral systems co-evolve, governed by competition and natural selection. Evolution leads to the emergence of a simple and robust social norm, which the authors call *stern-judging,* where implacable punishment goes side-by-side with prompt forgiving. The low level of complexity of this norm, which is supported by empirical observations in e-trade, conveys the idea that simplicity is often associated with evolutionary success.

## Introduction

Many biological systems employ cooperative interactions in their organization [1]. Humans, unlike other animal species, form large social groups in which cooperation among non-kin is widespread. This contrasts with the general assumption that the strong and selfish individuals are the ones who benefit most from natural selection. This being the case, how is it possible that unselfish behaviour has survived evolution? Adopting the terminology resulting from the seminal work of Hamilton, Trivers, and Wilson [2–4], an act is altruistic if it confers a benefit ***b*** to another individual in spite of accruing a cost ***c*** to the altruist (where it is assumed, as usual, that ***b > c***). In this context, several mechanisms have been invoked to explain the evolution of altruism, but only recently an evolutionary model of indirect reciprocity (using the terminology introduced by [5]) has been developed by Nowak and Sigmund [6], which, with remarkable simplicity, addressed “unique aspects of human sociality, such as trust, gossip, and reputation” [7]. As a means of community enforcement, indirect reciprocity had been investigated earlier in the context of economics, notably by Sugden [8] and Kandori [9] (see below). More recently, many studies [7,8,10–17] have been devoted to investigating how altruism can evolve under indirect reciprocity. Indeed, according to Alexander [5], indirect reciprocity presumably provides the mechanism that distinguishes us humans from all other living species on Earth. Moreover, as recently argued in [10], “indirect reciprocity may have provided the selective challenge driving the cerebral expansion in human evolution.” In the indirect reciprocity game, any two players are supposed to interact at most once with each other, one in the role of a potential donor, with the other as a potential receiver of help. Each player can experience many rounds, but never with the same partner twice, direct retaliation being unfeasible. By helping another individual, a given player may increase (or not) her reputation, which may change the predisposition of others to help her in future interactions. However, her new reputation depends on the social norm used by her peers to assess her action as a donor. Previous studies of reputation-based models of cooperation reviewed recently [10] indicate that cooperation outweighs defection whenever, among other factors, assessment of actions is based on norms that require considerable cognitive capacities [10,12,13], even when individuals are capable of making binary assessments only, in a “world in black and white” [10], as assumed in most recent studies (see, however, [6]). Furthermore, stable cooperation hinges on the availability of reliable reputation information [6]. Such high cognitive capacity contrasts with technology-based interactions, such as e-trade, which also rely on reputation-based mechanisms of cooperation [18–20]. Indeed, anonymous one-shot interactions between individuals loosely connected and geographically dispersed usually dominate e-trade, raising issues of trust-building and moral hazard [21]. Reputation in e-trade is introduced via a feedback mechanism which announces the ratings of sellers. Despite the success and high levels of cooperation observed in e-trade, it has been found [18] that publicizing a detailed account of the seller's feedback history does not improve cooperation, as compared with publicizing only the seller's most recent rating. In other words, practice shows that simple reputation-based mechanisms are capable of promoting high levels of cooperation. In view of the previous discussion, it is hard to explain the success of e-trade on the basis of the results obtained so far for reputation-based cooperation in the context of indirect reciprocity.

### A Model of Multilevel, Multigame Selection

Let us consider a world in black and white consisting of a set of tribes, such that each tribe lives under the influence of a single norm, common to all individuals (see Figure 1). Each individual engages once in the indirect reciprocity game (cf. Methods) with all other tribe inhabitants. Her action as a donor will depend on her individual strategy, which dictates whether she will *provide help* or *refuse to do it* depending on her and the recipient's reputation. Reputations are public: this means that the result of every interaction is made available to everyone through the “indirect observation model” introduced in [13] (see also [15]). This allows any individual to know the current status of the co-player without observing all of her past interactions. On the other hand, this requires a way to spread the information (even with errors) to the entire population (communication/language). Consistently, language seems to be an important cooperation promoter [22], although recent mechanisms of reputation-spreading rely on electronic databases (e.g., in e-trade, where reputation of sellers is centralized). Since reputations are either *GOOD* or *BAD,* there are ***2^4^ = 16*** possible strategies. On the other hand, the number of possible norms depends on their associated order. The simplest are the so-called *first-order norms,* in which all that matters is the action taken by the donor. In *second-order norms,* the reputation of one of the players (donor or recipient) also contributes to decide the new reputation of the donor. And so on, in increasing layers of complexity (and associated requirements of cognitive capacities from individuals) as shown in Figure 2, which illustrates the features of third-order norms such as those we shall employ here. Any individual in the tribe shares the same norm, which in turn raises the question of how each inhabitant acquired it. We do not address this issue here. However, inasmuch as indirect reciprocity is associated with “community enforcement” [9,10], one may assume, for simplicity, that norms are acquired through an educational process. Moreover, it is likely that a common norm contributes to the overall cohesiveness and identity of a tribe. It is noteworthy, however, that if norms were different for different individuals, the “indirect observation model” would not be valid, as it requires trust in judgments made by co-inhabitants. For a norm of order ***n*** *,* there are 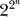 possible norms, each associated with a binary string of length 2*^n^*. We consider third-order norms (8-bit strings, Figure 2): in assessing a donor's new reputation, the observer has to make a contextual judgment involving the donor's action, as well as her and the recipient's reputations scored in the previous action. We introduce the following evolutionary dynamics in each tribe: during one generation all individuals interact once with each other via the indirect reciprocity game. When individuals “reproduce,” they replace their strategy by that of another individual from the same tribe, chosen proportional to her accumulated payoff [12]. The most successful individuals in each tribe have a higher reproductive success. Since different tribes are “under the influence” of different norms, the overall fitness of each tribe will vary from tribe to tribe, as well as the plethora of successful strategies that thrive in each tribe (Figure 1). This describes individual selection in each tribe (Level 1 in Figure 1).

**Figure 1:**
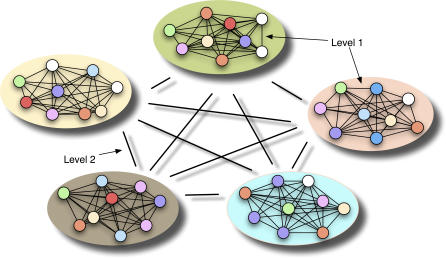
Multilevel Selection Model for the Evolution of Norms Each palette represents a tribe in which inhabitants (coloured dots) employ different strategies (different colours) to play the indirect reciprocity game. Each tribe is influenced by a single social norm (common background colour), which may be different in different tribes. All individuals in each tribe undergo pairwise rounds of the game (lower level of selection, Level 1), whereas all tribes also engage in pairwise conflicts (higher level of selection, Level 2), as described in the main text. As a result of the conflicts between tribes, norms evolve, whereas evolution inside each tribe selects the distribution of strategies that best adapt to the ruling social norm in each tribe.

**Figure 2:**
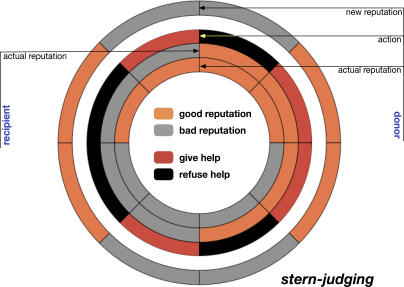
Norm Complexity The higher the order (and complexity) of a norm, the more “inner” layers it acquires. The outer layer stipulates the donor's new reputation based on the three different reputation/action combinations aligned radially layer by layer: inward, the first layer identifies the action of the donor. The second layer identifies the reputation of the recipient; the third the reputation of the donor. Out of the 2^8^ possible norms, the highly symmetric norm shown as the outer layer emerges as the most successful norm. Indeed, stern-judging renders the inner layer (donor reputation) irrelevant in determining the new reputation of the donor. This can be trivially confirmed by the symmetry of the figure with respect to the equatorial plane (not taking the inner layer into account, of course). All norms of second order will exhibit this symmetry, although the combinations of 1 and 0 bits will be, in general, different. We use this representation in Protocol S1 to depict other popular norms, namely, the *leading-eight, standing, simple-standing,* and *image-scoring*.

Tribes engage in pairwise conflicts with a small probability, associated with selection between tribes. After each conflict, the norm of the defeated tribe will change toward the norm of the victor tribe, as detailed in the Methods section (Level 2 in Figure 1). We consider different forms of conflict between tribes, which reflect different types of inter-tribe selection mechanisms: *group selection* [5,23–28] based on the average global payoff of each tribe (involving different selection processes and intensities; imitation dynamics, a Moran-like process; and the pairwise comparison process, the latter discussed in Protocol S1) as well as selection resulting from inter-tribe conflicts modeled in terms of games—the display game of war of attrition, and an extended hawk–dove game [14] (see Protocol S1). We perform extensive computer simulations of evolutionary dynamics of sets of *64* tribes, each with *64* inhabitants. Once a stationary regime is reached, we collect information for subsequent statistical analysis (cf. Methods). We compute the frequency of occurrence of bits ***1*** and ***0*** in each of the 8-bit locations. A bit is said to fixate if its frequency of occurrence exceeds or equals *98%*. Otherwise, no fixation occurs, which we denote by ***X***, instead of by ***1*** or ***0***. We analyze *500* simulations for the same value of ***b*** *,* subsequently computing the frequency of occurrence ***ϕ_1_*** *,* ***ϕ_0_,*** and ***ϕ_X_*** of the bits ***1*** *,* ***0*** *,* and ***X*** *,* respectively. If ***ϕ_1_ >*** ***ϕ_0_*** ***+*** ***ϕ_X_*** *,* the final bit is ***1*** *;* if ***ϕ_0_ >*** ***ϕ_1_*** ***+*** ***ϕ_X_*** *,* the final bit is ***0*** *;* otherwise we assume it is indeterminate, and denote it by •. It is noteworthy that our bit-by-bit selection/transmission procedure, though artificial, provides a simple means of mimicking biological evolution, where genes are interconnected by complex networks and yet evolve independently. Certainly, a co-evolutionary process would be more appropriate (and more complex), and this will be explored in future work.

## Results/Discussion

The results for different values of ***b*** are given in Table 1, showing that a unique, ubiquitous social norm emerges from these extensive numerical simulations. This norm is of *second-order,* which means that all that matters is the action of the donor and the reputation of the receiver. In other words, even when individuals are equipped with higher cognitive capacities, they rely on a simple norm as a key for evolutionary success. In a nutshell, helping a good individual or refusing help to a bad individual leads to a good reputation, whereas refusing help to a good individual or helping a bad one leads to a bad reputation. Moreover, we find that the final norm is independent of the specifics of the second-level selection mechanism, i.e., different second-level selection mechanisms will alter the rate of convergence, but not the equilibrium state. In this sense, we conjecture that more realistic procedures will lead to the same dominant norm.

**Table 1:**
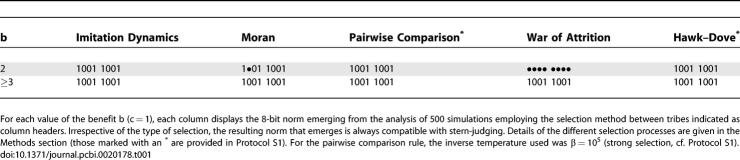
Emerging Social Norm

The success and simplicity of this norm relies on never being morally dubious: to each type of encounter, there is one *GOOD* move and one *BAD* one. Moreover, it is always possible for anyone to be promoted to the best standard possible in a single move. Conversely, one bad move will be readily punished [29,30] with the reduction of the player's score. This prompt forgiving and implacable punishment leads us to call this norm *stern-judging*.

Long before the seminal work of Nowak and Sigmund [6], several social norms have been proposed as a means to promote (economic) cooperation. Notable examples are the *standing* norm, proposed by Sugden [8], and the norm proposed by Kandori [9], as a means to allow community enforcement of cooperation. When translated into the present formulation, *standing* constitutes a third-order norm, whereas a fixed-order reduction of the social norm proposed by Kandori (of variable order, dependent on the benefit-to-cost ratio of cooperation) would correspond to *stern-judging.* Indeed, in the context of community enforcement, one can restate *stern-judging* as: “Help good people and refuse help otherwise, and we shall be nice to you; otherwise, you will be punished.”

It is, therefore, most interesting that the exhaustive search carried out by Ohtsuki and Iwasa [13,15] in the space of up to third-order norms found that these two previously proposed norms were part of the so-called *leading-eight* norms of cooperation. On the other hand, *image-score,* the norm emerging from the work of Nowak and Sigmund [6], which has the attractive feature of being a simple, second-order norm (like *stern-judging*) does not belong to the *leading-eight*. Indeed, the features of *image-scoring* have been carefully studied in comparison with *standing* [7,16,17], showing that *standing* performs better than *image-scoring,* mostly in the presence of errors [12].

Among the *leading-eight* norms discovered by Ohtsuki and Iwasa [13,15], only *stern-judging* [10] and the so-called *simple-standing* [31] constitute second-order norms (see below). Our present results clearly indicate that *stern-judging* is favored compared with all other norms. Nonetheless, in line with the model considered here, the performance of each of these norms may be evaluated by investigating how each norm performs individually, *taking into account all 16 strategies simultaneously*. In Protocol S1, we carry out such comparison: we consider *standing* among the third-order norms, as well as *stern-judging, image-scoring,* and *simple-standing* among second-order norms. Note that, in spite of fixating the norm, errors of assessment and of implementation as well as errors of strategy update are taken into account. The results show that the overall performance of *stern-judging* is better than that of the other norms over a wide range of values of the benefit ***b***. Furthermore, both *standing* and *simple-standing* perform very similarly, again pointing out that reputation-based cooperation can successfully be established without resorting to higher-order (more sophisticated) norms. Finally, *image-scoring* performs considerably worse than all the other norms, a feature already addressed [7,16,17]. Within the space of second-order norms, similar conclusions have been found recently by Ohtsuki and Iwasa [31]. Note, however, that the result obtained here is stronger than the analysis carried out in Protocol S1, since *stern-judging* emerges as the most successful norm surviving selection and mutation with other norms, irrespective of the selection mechanism. In other words, *stern-judging*'s simplicity and robustness to errors may contribute to its evolutionary success, since other well-performing strategies may succumb to invasion of individuals from other tribes who bring along strategies that may affect the overall performance of a given tribe. In this sense, robustness plays a key role when evolutionary success is at stake. We believe that *stern-judging* is the most robust norm promoting cooperation.

The present result correlates nicely with the recent findings in e-trade, where simple, reputation-based mechanisms ensure high levels of cooperation. Indeed, *stern-judging* involves a straightforward and unambiguous reputation assessment, decisions of the donor being contingent only on the previous reputation of the receiver. We argue that the absence of constraining environments acting upon the potential customers in e-trade, for whom the decision of buying or not buying is free from further ado, facilitates the adoption of a *stern-judging* assessment rule. Indeed, recent experiments [32] have shown that humans are very sensitive to the presence of subtle, psychologically constraining cues, their generosity depending strongly on the presence or absence of such cues. Furthermore, under simple unambiguous norms, humans may escape the additional costs of conscious deliberation [33].

As conjectured by Ohtsuki and Iwasa [13] (cf. also [5,23]), group selection might constitute the key element in establishing cooperation as a viable trait. The present results show that even when more sophisticated selection mechanisms operate between tribes, the outcome of evolution still favors *stern-judging* as the most successful norm under which cooperative strategies may flourish.

## Materials and Methods

We considered sets of *64* tribes, each tribe with *64* inhabitants. Each individual engages in a single round of the following indirect reciprocity game [10] with every other tribe inhabitant, assuming with equal probability the role of donor or recipient. The donor decides *YES* or *NO,* if she will provide help to the recipient, following her individual strategy encoded as a 4-bit string [12–14] (which takes into account the current donor and recipient status—see Protocol S1). If *YES*, then her payoff decreases by ***1*** *,* while the recipient's payoff increases by ***b > 1***. If *NO*, the payoffs remain unchanged (following common practice [6,12,14,16,21], we increase the payoff of every interacting player by ***1*** in every round to avoid negative payoffs). This action will be witnessed by a third-party individual, who, based on the tribe's social norm, will ascribe (subject to some small error probability ***μ_a_*** = 0.001) a new reputation to the *donor,* which we assume to spread efficiently without errors to the rest of the individuals in that tribe [12–14]. Moreover, individuals may fail to do what their strategy compels them to do, with a small execution error probability ***μ_e_*** = 0.001. After all interactions take place, one generation has passed, simultaneously for all tribes. Individual strategies in each tribe replicate to the next generation in the following way: for every individual ***A*** in the population, we select an individual ***B*** proportional to fitness (including ***A***) [12]. The strategy of ***B*** replaces that of ***A*** *,* apart from bit mutations occurring with a small probability ***μ_s_*** = 0.01. Subsequently, with probability ***p_CONFLICT_ = 0.01*** *,* all pairs of tribes may engage in a conflict, in which each tribe acts as an individual unit. Different types of conflicts between tribes have been considered.

### Imitation selection.

We compare the average payoffs ***Π_A_*** and ***Π_B_*** of the two conflicting tribes ***A*** and ***B*** *,* the winner being the tribe with highest score.

### Moran process.

In this case the selection method between tribes mimics that used between individuals in each tribe; one tribe ***B*** is chosen at random, and its norm is replaced by that of another tribe ***A*** chosen proportional to fitness.

### War of attrition.

We choose at random two tribes ***A*** and ***B*** with average payoffs ***Π_A_*** and ***Π_B_***. We assume that each tribe can display its strength for a time, which is an increasing function of its average payoff. To this end, we draw two random numbers, ***R_A_*** and ***R_B_***, each following an exponential probability distribution given by *exp*(*−t/**Π_A_*** )/***Π_A_*** and *exp*(*−t/**Π_B_*** )/***Π_B_***, respectively. The larger of the two numbers identifies the winning tribe.

As a result of inter-tribe conflict (two additional conflicts are discussed in Protocol S1), the norm of the losing tribe (***B***) is shifted in the direction of the victor norm (***A***). Convergence of such a nonlinear evolutionary process dictates a smooth norm crossover. Hence, each bit of norm***A*** will replace the corresponding bit of norm***B*** with probability

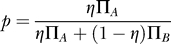
which ensures good convergence whenever *η* ≤ 0.2, independently of the type of conflict (a bit mutation probability *μ_N_* = 0.0001 has been used). Furthermore, a small fraction of the population of tribe ***A*** replaces a corresponding random fraction of tribe ***B*** *:* each individual of tribe ***A*** replaces a corresponding individual of tribe ***B*** with a probability *μ_migration_ = 0.005*. Indeed, if no migration takes place, a tribe's population may get trapped in less cooperative strategies, compromising the global convergence of the evolutionary process [26].

Each simulation runs for *9,000* generations, starting from randomly assigned strategies and norms, to let the system reach a stationary situation, typically characterized by all tribes having maximized their average payoff, for a given benefit ***b > c = 1***. The subsequent *1,000* generations are then used to collect information on the strategies used in each tribe and the norms ruling the tribes in the stationary regime. We ran *500* evolutions for each value of ***b*** *,* subsequently performing a statistical analysis of the bits that encode each norm, as detailed before.

The results presented are quite robust to variations of the different mutation rates introduced above, as well as to variation of population size and number of tribes. Furthermore, reducing the threshold from *98%* to *95%* does not introduce any changes in the results shown.

## Supporting Information

Protocol S1 Click here for additional data file. Supplementary Information
(1.0 MB DOC)
