# Supplementary material for: Stern-Judging: A Simple, Successful Norm Which Promotes Cooperation under Indirect Reciprocity
Source: PLoS Comput Biol. 2006 Dec 29;2(12):e178. doi: 10.1371/journal.pcbi.0020178 (PMC1761656; doi:10.1371/journal.pcbi.0020178)
Supplement: Supplementary file 1 [file pcbi.0020178.sd001.doc]

**SUPPLEMENTARY INFORMATION**

Stern-judging : A simple, successful norm which promotes cooperation under indirect reciprocity
J. M. Pacheco^1^, F. C. Santos^2^ and F. A. C. C. Chalub^3 *^

**Simulation details**

In our simulations, we adopted the following values: *η*=0.1, *μ*_N_=0.0001, *μ*_S_=0.01, *μ*_a_=*μ*_e_=0.001. The benefit ***b*** varied from ***b*** =2 to ***b***=36.

We ran each simulation for *9000* generations and computed the average using the subsequent *1000* results. As a cross validation, results did not change if instead we ran simulations for *14000* generations, accumulating information over the subsequent *1000* generations. This indicates that a steady state has been reached. Finally, results are robust to reasonable changes of the parameters above.

Each individual, in each tribe, has a strategy (chosen randomly at start) encoded as a four-bit string, which determines the individual’s action (**N**=no, do not provide help; **Y**=yes, provide help) as a donor, knowing hers and the recipient’s reputation, as detailed in Table S1. This results in a total of 16 strategies, ranging from unconditional defection (***ALLD***) to unconditional cooperation (***ALLC***), as detailed in Table S2. These two extreme strategies are however, norm-independent. Hence, our statistical analysis only takes into account those steady states in which the prevalence of any of these strategies is below a given threshold. The results shown correspond to a maximum threshold of 10%, although results did not change by reducing or increasing this threshold by a factor of two.

**Pairwise comparison and norm evolution for different intensities of selection**

The pairwise comparison rule [1] provides a convenient framework to study how the intensity of selection between tribes affects the emergence of stern-judging. It corresponds to introduce the following dynamics: Given two tribes chosen for a conflict, say ***A*** and ***B***, with average payoffs ***_A_*** and ***_B_***, respectively, then norm of tribe ***B*** will replace that of ***A*** with a probability given by

$$p=\left[ 1+e^{-\beta(\Pi_{B}-\Pi_{A})} \right]^{-1}$$

whereas the inverse process will occur with probability $(1-p)$. In physics this function corresponds to the well-known Fermi distribution function, in which the inverse temperature$\beta$ determines the sharpness of transition from$p=0$, whenever***_B_ <*** ***_A_***, to $p=1$, whenever ***_A_ <_B_***. Indeed, in the limit $\beta\to+\infty$ we obtain imitation dynamics (strong selection), whereas whenever $\beta\to0$ ***B*** replaces ***A*** with the same probability that ***A*** replaces ***B*** ( ½ - neutral drift). As we change$\beta$ between these two extreme limits, we can infer the role of selection intensity on the emergence of stern-judging. In Table S3 we show results for different values of$\beta$, which testify for the robustness of stern-judging. In other words, in spite of the fact that, with decreasing$\beta$ (decreasing selection intensity), it becomes increasingly difficult for all 8 bits to fixate whenever ***b=2***, in no case do we get results which deviate from stern-judging as the emerging social norm. These results (together with the analysis carried out in the following for inter-tribe selection determined by a Hawk-Dove game), reinforce the conclusion that stern-judging is robust and ubiquitous.

**Hawk-Dove Tribal Conflict**

This method of tribal conflict has been developed in Ref. [2] and is based on an extended Hawk-Dove game introduced in Ref. [3]. If tribe ***A*** goes to war, then we choose at random its adversary (***B***) from the remaining tribes. Average payoffs of both tribes are denoted, as usual, by $\Pi_{A}$ and $\Pi_{B}$respectively.

For each tribe there are two possible strategies, HAWK and DOVE, similar to the Hawk-and-Dove game described in [3]. The payoff matrix (for player ***A***) reads

|  | **DOVE** | **HAWK** |
| --- | --- | --- |
| **DOVE** | **V**/2 - **T** | 0 |
| **HAWK** | **V** | (**V**-**W**)*p*-**L**(1-*p*) |

where *p*(***_A_***,***_B_***) = *p*(***_A_***-***_B_***) is the probability that ***A*** wins a contest against ***B*** (estimated by ***A***) when both play HAWK with given average payoff. In particular, we shall adopt *p*=*p_β_* (x) = [1+ exp(-βx)]^-1^, where the inverse temperature β>0 is assumed to be the same for all tribes. The most interesting scenario [3] occurs whenever **L**>**W**>0, **V**>**W**>0, **L**+**W**>**V**>2**T**>0 and, in order to avoid negative payoffs, we add the absolute value of the minimum possible payoff, **L**, to all players after one conflict, a procedure which does not introduce any changes in the game. Hence we adopted the values **V**=1, **T**=0.01, **W**=1/ 2, **L**=3/4 and β=10^4^.

We assume that tribes are rational players, such that tribe ***A*** will play HAWK with probability *q*(*p_θ_* (***_A_***-***_B_***)) associated with the Nash equilibrium of the game's payoff matrix. Defining *r*:= (**V**-**W**+**L**)*p*-**L** we have *q*(*p*)=1 if *r*=0, and *q*(*p*)=[1-*r*/(**V**/2+**T**)]^-1^ otherwise. Similarly, tribe ***B*** will play HAWK with probability *q*(*p_θ_* (***_B_***-***_A_***)) =*q***(**1**-** *p_θ_* (***_A_***-***_B_***)). After conflict, the norms adopted by tribes ***A*** and ***B*** will possibly change from what they were before. Let **Q(*A*)** be the payoff obtained by ***A*** and **Q(*B*)** that obtained by ***B*** as a result of the game. Then:

- If **A** played HAWK and **Q(*A*)** > **Q(*B*)**, then each bit of norm of ***B*** will change with the probability defined in the methods section, incuding a mutation probability *μ*_N_.
- Same as before, swapping ***A*** and ***B***.
- If ***A*** played HAWK and **Q(*A*)** < **Q(*B*)** or ***A*** played DOVE, then norm entries N**_B_**(i) are mutated with probability *μ*_N_«1 and the population strategies are mutated by *μ*_S_.
- Same as before, swapping ***A*** and ***B***.

Results for this update rule, shown in Table 1, provide clear evidence for the robustness and ubiquity of stern-judging. In this case, we obtain fixation of bits even for values of *b < 3*.

**Cooperation under selected social norms**

In order to better understand the success of ***stern-judging***, we carry out in the following a study of how tribes perform under the influence of a specific norm *which we now fix from the outset*. We shall compare the performance of ***stern-judging*** with the popular norms *standing* and *image-scoring*, as well as with the other second-order norm which incorporates the leading eight, coined strict standing [4]. We shall maintain mutation errors in strategy update, as well as errors of implementation. As a result, and given a fixed (immutable) norm, selection and mutation dictates the simultaneous evolution of all the 16 strategies in a given tribe. We are not aware of any study which undertook such a comparison. Indeed, in Ohtsuki and Iwasa’s seminal work [5], they searched for well defined combinations of one norm which would constitute a non-trivial Evolutionary Stable Strategy in a monomorphic population with an associated cooperative strategy. Hence they discovered the leading eight. In Fig. S1 we depict the leading eight norms, using the convention of Fig. 2. The white “slices” correspond to places where both *GOOD* (orange) or *BAD* (grey) reputations can be freely assigned, the remaining norm being on of the leading eight. Since a second order norm, in this representation, is simply a norm which exhibits a mirror symmetry with respect to the equatorial plane, it is obvious that there are only two second order norms which incorporate the leading eight: Besides ***stern-judging***, also *simple-standing* belongs to the leading eight. Both norms form the first row of Fig. S2, whereas image-scoring and standing, the original norm proposed by Sugden, complete the lower row in Fig. S2.

Brandt and Sigmund [6] have carried an individual based model analysis in which evolution took place under selection and mutation between individuals whose norm (in the sense defined here) was individually assigned, as well as the strategy. Moreover, information was private, not public. Finally, Ohtsuki and Iwasa have recently [4] examined which strategies thrive under the presence of a single, second-order norm, now in a (infinite) polymorphic population in which individuals can adopt three out of the 16 strategies considered in this work. Their analytic study leads to the conclusion that, in the presence of errors, stable coexistence between conditional and unconditional cooperators is possible, ***stern-judging*** constituting one of the leading norms promoting cooperative behaviour.

In Fig. S3 we show results for the ratio between the average payoff reached in each tribe and the maximum average payoff attainable in that tribe, given the tribe size and the benefit (keeping cost=1). This quantity is plotted as a function of the benefit from cooperation, *b.* The results in Fig. S3 show that ***stern-judging*** performs better than any of the other norms. Both *standing* and *simple-standing* lead to very similar performance, which reinforces the idea that second order norms are enough to promote cooperation under indirect reciprocity. Finally, image-scoring performs poorly compared to any of the other norms, a feature which is also related to the fact that the present analysis was carried out in the presence of errors.
The marginal advantage of ***stern-judging***, obtained via the present analysis, may not be enough to justify its ubiquity and insensitivity with respect to the mechanisms of selection between tribes as well as to the intensity of selection between tribes. We believe that, besides its excellent overall performance, ***stern-judging*** is more robust to invasion by other strategies, which gives it an evolutionary advantage with respect to other successful norms which promote cooperation.

**References**

1. Traulsen A, Nowak MA, Pacheco JM ((in press)) Stochastic dynamics of invasion and fixation. Physical Review E

2. Chalub FACC, Santos FC, Pacheco JM (2006) The evolution of norms. J Theor Biol 241: 233-240.

3. Crowley PH (2000) Hawks, doves, and mixed-symmetry games. J Theor Biol 204: 543-563.

4. Ohtsuki H, Iwasa Y (submitted) Global analysis of evolutionary dynamics and exhaustive search for social norms that maintain cooperation and reputation.

5. Ohtsuki H, Iwasa Y (2004) How should we define goodness?--reputation dynamics in indirect reciprocity. J Theor Biol 231: 107-120.

6. Brandt H, Sigmund K (2004) The logic of reprobation: assessment and action rules for indirect reciprocation. J Theor Biol 231: 475-486.

| ***donor*’s**  ***reputation*** | ***Recipient*’s reputation** | ***donor*’s**  **action** |
| --- | --- | --- |
| *GOOD* | *GOOD* | **Y**  / **N** |
| *GOOD* | *BAD* | **Y**  / **N** |
| *BAD* | *GOOD* | **Y**  / **N** |
| *BAD* | *BAD* | **Y**  / **N** |

**Table S1. Bit-encoding of individual strategies.** Each individual has a strategy encoded as a four-bit string. For each combination pair of donor and recipient reputations, the strategy prescribes individual’s action. There are a total of 2^4^=16 strategies, identified in Table S2.

| ***strategy name*** | ***GG*** | ***GB*** | ***BG*** | ***BB*** |
| --- | --- | --- | --- | --- |
| **ALLD** | **N** | **N** | **N** | **N** |
| **1** | **N** | **N** | **N** | **Y** |
| **AND** | **N** | **N** | **Y** | **N** |
| **SELF** | **N** | **N** | **Y** | **Y** |
| **4** | **N** | **Y** | **N** | **N** |
| **5** | **N** | **Y** | **N** | **Y** |
| **6** | **N** | **Y** | **Y** | **N** |
| **7** | **N** | **Y** | **Y** | **Y** |
| **8** | **Y** | **N** | **N** | **N** |
| **9** | **Y** | **N** | **N** | **Y** |
| **CO** | **Y** | **N** | **Y** | **N** |
| **OR** | **Y** | **N** | **Y** | **Y** |
| **12** | **Y** | **Y** | **N** | **N** |
| **13** | **Y** | **Y** | **N** | **Y** |
| **14** | **Y** | **Y** | **Y** | **N** |
| **ALLC** | **Y** | **Y** | **Y** | **Y** |

**Table S2. Different individual strategies in indirect reciprocity game.** We identify the different strategies and how they determine the action of a donor (**N**=no, do not provide help, **Y**=yes, provide help), given the reputation pair donor/recipient. Whereas some of these strategies have assumed well-known designations in the literature, others remain named by their numeric order. This convention has been adopted in Fig. S1.

| ***b*** | $\beta=\text{10}^{5}$ | $\beta=\text{10}^{4}$ | $\beta=\text{10}^{3}$ | $\beta=\text{10}^{2}$ | $\beta=\text{10}^{1}$ | $\beta=\text{10}^{0}$ |
| --- | --- | --- | --- | --- | --- | --- |
| 2 | 1001 1001 | 1●01 1001 | 1●01 100● | 1●01 100● | 1●01 100● | ●●●● ●●●● |
| $3$ | 1001 1001 | 1001 1001 | 1001 1001 | 1001 1001 | 1001 1001 | 1001 1001 |

**Table S3. Emergence of stern-judging for different intensities of selection.** We carried out the bit-fixation analysis described in main text for the evolution of social norms under the pairwise comparison rule, for different values of the intensity of selection$\beta$. Intensity of selection decreases from left to right. Whereas for strong selection all norm bits fixate for b≥2, fixation becomes more difficult for b=2 as $\beta$ decreases. Yet, in no case did we obtain fixation of a digit incompatible with stern-judging.

**Figure S1. The Leading Eight Norms of Ohtsuki and Iwasa**. We use the same notation as in Fig. 2, leaving in white those “slices” in the final norm which can be associated with either *GOOD* (orange) or *BAD* (grey) reputations. Note that, in this convention, second order norms exhibit a mirror symmetry with respect to the equatorial plane (disregarding the innermost layer). As a result, only two second order norms can incorporate the leading-eight – ***stern-judging*** and *simple-standing*, as recently coined by Ohtsuki and Iwasa – see Fig. S2 for details.

**Figure S2. Four norms which promote cooperation.** We depict the four norms, the performance of which we analysed. Both ***stern-judging***, *simple-standing* and *image-scoring* are symmetric with respect with the equatorial plane, and as such are second order norms. As for *standing*, it clearly breaks this symmetry, constituting a third order norm. In this representation, it is also clear that ***stern-judging***, exhibits the simplest symmetry of all norms.

**Figure S3. Individual performance of norms.** We plot the ratio between the average payoff attained by each tribe under the influence of a single, fixed norm, and the maximum value possible, given the population size (64), the benefit from cooperation (*b*) and the cost of cooperation (*c=1*). Overall, ***stern-judging*** performs better than the other three norms of cooperation considered (cf. Fig. S2). For small values of *b,* the advantage is smaller than for larger values, but it is never superseded by any other norm. It is remarkable that *standing*, a third order norm, performs almost as well as *simple-standing*, a simpler, second-order norm. Finally, in all cases *image-score* is unable to match the performance of the other three norms. We ran 500 simulations for each tribe with 64 inhabitants, and used the last 1000 generations from a total of 10000 in each simulation to compute the average values depicted. We have included errors of execution as well as mutation of strategies.
